# Osteosarcoma of the Pelvis: Clinical Presentation and Overall Survival

**DOI:** 10.1155/2021/8027314

**Published:** 2021-12-06

**Authors:** Jeffrey Mark Brown, David Matichak, Kyla Rakoczy, John Groundland

**Affiliations:** ^1^University of Miami Miller School of Medicine, Miami, FL, USA; ^2^Department of Orthopaedics, Huntsman Cancer Institute, University of Utah School of Medicine, Salt Lake City, UT, USA

## Abstract

**Introduction:**

Osteosarcoma is the most common sarcoma of bone. Pelvic osteosarcoma presents a significant therapeutic challenge due to potential late symptom onset, metastatic dissemination at diagnosis, and inherent difficulties of wide surgical resection secondary to the complex and critical anatomy of the pelvis. The rates of survival are well reported for osteosarcoma of the appendicular skeleton, but specific details regarding presentation and survival are less known for osteosarcoma of the pelvis.

**Methods:**

The Surveillance, Epidemiology, and End Results (SEER) program was queried for primary osteosarcoma of the bony pelvis from 2004 to 2015. Cases with Collaborative Staging variables (available after 2004) were analyzed by grade, histologic subtype, surgical intervention, tumor size, tumor extension, and presence of metastasis at diagnosis. The 2-, 5-, and 10-year survival rates were assessed with respect to these variables. The SEER database was then queried for age, tumor size, surgical intervention, metastasis at time of presentation, and survivorship data for patients with primary osteosarcoma of the upper extremity, lower extremity, vertebrae, thorax, and face/skull, and rates for all anatomic locations were then compared to patients with primary pelvic osteosarcoma.

**Results:**

A total of 292 cases of pelvic osteosarcoma were identified from 2004 to 2015 within the database, representing 9.8% of cases among all surveyed primary sites. The most common histologic subtype was osteoblastic osteosarcoma (69.9%), followed by chondroblastic osteosarcoma (22.3%). The majority of cases were high-grade tumors (94.3%), of size >8 cm (72.0%), and with extension beyond the originating bone (74.0%). For the entire pelvic osteosarcoma group, the 2-, 5-, 10-year survival rates were 45.6%, 26.5%, and 21.4%, respectively, which were the poorest among surveyed anatomic sites. The 5-year overall survival was an abysmal 5.3% for patients with metastatic disease at diagnosis, and 37.0% for non-metastatic pelvic osteosarcoma treated with surgery and chemotherapy. When compared to other locations, pelvic osteosarcoma had higher rates of metastatic disease at presentation (33.5%), larger median tumor size (11.0 cm), and older median age at diagnosis (47.5 years). While over 85% of patients with tumors at the extremities received surgery, only 47.4% of pelvic osteosarcomas in this cohort received surgical resection—likely influenced by larger tumor size, sacral involvement, frequency of metastasis, older age, or delayed referral to a sarcoma center.

**Conclusion:**

This study clarifies presenting features and clinical outcomes of pelvic osteosarcomas, which often present with large, high-grade tumors with extracompartmental extension, high likelihood of metastatic disease at diagnosis, and a potential limited ability to be addressed surgically. The survival rates of primary osteosarcoma of the pelvis are poor and are lower than osteosarcomas from other anatomic locations. While acknowledging the influence of metastasis, tumor characteristics, and advanced age on the decision to undergo surgical excision of a pelvic osteosarcoma, the rates of surgical resection are low and highlight the importance of understanding appropriate conditions for oncologic resection of pelvic sarcomas.

## 1. Introduction

Osteosarcoma is the most common primary sarcoma of the bone[1–4]. The current standard of care for osteosarcoma includes an extended course of multiagent, cytotoxic chemotherapy and, whenever possible, wide surgical resection of the tumor [6]. With this course of treatment, the overall 5-year survival rate for osteosarcoma is most often reported between 60–70% [4, 7–10]. However, certain factors are known to strongly influence these survival rates. Most notably, the presence or absence of metastatic disease at presentation has profound implications on survival. Patients with no evidence of metastatic disease on presentation have improved survival (70–75% 5-year survival), but patients with distant metastases have a much poorer prognosis (25–35% 5-year survival) [4, 8, 9, 11–13]. While metastasis may be the most important factor known to influence the prognosis of osteosarcoma, other variables may have effects as well [4, 14, 15]. The impact of the primary site of osteosarcoma on survival rates has been broached in the literature, but detailed assessment of specific anatomic sites has been limited.

While the majority of cases occur about the knee, osteosarcoma may affect any bone within the skeleton. Approximately 5–10% of osteosarcoma cases primarily originate in the bony pelvis [8, 16–20]. The literature indicates that pelvic osteosarcoma has a worse prognosis than osteosarcomas of the appendicular skeleton. Studies investigating the survival rates of primary pelvis osteosarcoma suggest that the 5-year survival rate is between 15–30% rather than the 60–70% rate reported for osteosarcoma as a whole [15, 17–27]. These studies give important insight into the overall prognosis of pelvic osteosarcoma, yet they are limited by sample size and potential institutional bias and were unable to assess variables that may influence presentation and outcome.

The primary questions of this study are as follows: what are the 2-, 5- and 10-year survival rates of pelvic osteosarcoma, and what factors influence survival? The factors investigated were patient age, histologic subtype, size of the tumor, local invasiveness, grade, metastatic presentation, and treatments rendered. The secondary questions of this study are as follows: what are the differences in metastasis on presentation, and what are the differences in the 2-, 5-, and 10-year survival rates between pelvic osteosarcoma and other anatomic sites of primary osteosarcoma? To maximize the number of patients available for analysis, the National Cancer Institute's Surveillance, Epidemiology, and End Results (SEER) database was used.

## 2. Methods

The National Cancer Institute's Surveillance, Epidemiology, and End Results (SEER) database was queried by year of diagnosis, histologic subtype, grade, and anatomic location to identify cases of primary osteosarcoma of the pelvic bones, sacrum, coccyx, and their associated joints (ICD site code C41.4). Cases of conventional/osteoblastic, chondroblastic, fibroblastic, telangiectatic, and Paget-associated osteosarcoma localized to the bony pelvis were included. The term “conventional” osteosarcoma used in this manuscript and the SEER data is assumed to refer largely to osteoblastic osteosarcoma, the most common histologic subtype, in contrast to chondroblastic or fibroblastic subtypes which are explicitly defined in the SEER database. The authors acknowledge possible limitations of histologic description in the SEER data, specifically for tumors described as “conventional.” These histologic subtypes corresponded to the respective ICD-0–3 codes 9180/3, 9181/3, 9182/3, 9183/3, and 9184/3.

Cases were grouped by age within one of three categories (age <25, age 25–60, and age >60) and were limited to the years of diagnosis 2004–2015 in order to permit case description by Collaborative Staging variables, as these variables are only available from the year 2004 to 2015. Years after 2015 were excluded as the reporting variables within the database changed, and we wished to minimize any discrepancies in which the data were reported. The variables queried were sex, age, grade, histologic subtype, tumor size, tumor extension, presence of metastasis at diagnosis, and therapeutic intervention. Tumor size was characterized as ≤8 cm or >8 cm. Tumor extension was categorized by confinement within the periosteum of the originating bone (contained), extension beyond the periosteum, infiltrating the adjacent bone or cartilage, and skip metastases. Therapeutic modalities including surgical intervention, radiation, and chemotherapy were queried. Surgical interventions were broadly categorized as either internal hemipelvectomy (resection with retention of the lower extremity), external hemipelvectomy (hindquarter amputation), unspecified surgical intervention, or none. Chi-square tests were used to assess difference of these groups. Survival for the previously described variables and categories was assessed at 2-, 5,- and 10-year intervals using Kaplan–Meier analysis with the SEER Stat software. Log-rank tests with pairwise analysis were performed on the Kaplan–Meier curves to assess for statistical differences between groups. A Cox proportional hazards model was generated to produce a multivariate assessment of the impact of the preceding variables on survival.

The SEER database was then queried for osteosarcoma of the upper extremity, lower extremity, vertebrae, thorax, and face/skull. The same 2004–2015 timeframe was used, and the rates of metastatic disease on presentation, median age, percentage receiving surgical intervention, and the 2-, 5-, and 10-year survival were obtained. The rates of metastasis and survival intervention were compared to those found in the pelvis osteosarcoma cohort, and tested for significance using Chi-square tests. Median age at diagnosis and median tumor size were compared between groups with Kruskal–Wallis tests. Log-rank tests with pairwise analysis were performed on the Kaplan–Meier curves to assess for statistical differences between groups for survival.

## 3. Results

### 3.1. Presentation of Pelvis Osteosarcoma

**Figure 1:**
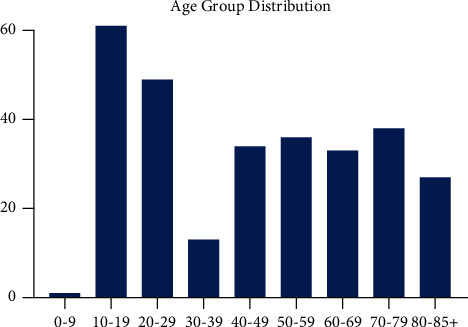
Age group histogram for osteosarcoma of the pelvis. A bimodal distribution is observed in adolescents and older adults.

Between 2004 and 2015, the SEER database has a total of 292 cases of primary osteosarcoma localized to the pelvis. The sex distribution was equal, with 149 male patients and 143 female patients across all histologic subtypes. Age at diagnosis ranged from 7 to 89, demonstrating a bimodal distribution, with an overall median age of 47.5 years at diagnosis (Figure 1). These findings and other presenting features are summarized in Table 1.

Osteoblastic or conventional osteosarcoma was the most common subtype (*n* = 204, 69.9%), followed by chondroblastic osteosarcoma (*n* = 65, 22.3%), Paget-associated osteosarcoma (*n* = 6, 2.1%), and fibroblastic osteosarcoma (*n* = 7; 2.4%); telangiectatic, parosteal, periosteal, and small cell osteosarcomas had 3 or fewer cases each (<1%). The median age of diagnosis for conventional osteosarcoma was 53.5 years. Chondroblastic osteosarcoma manifested in a significantly younger patient population than conventional osteosarcoma, with a median age at diagnosis of 22 years (*p* < 0.001). Paget-associated osteosarcoma presented in an older population, with a median age of diagnosis of 67.5 years, which was not significantly different from that of conventional osteosarcoma in this cohort (*p*=0.119).

**Figure 2:**
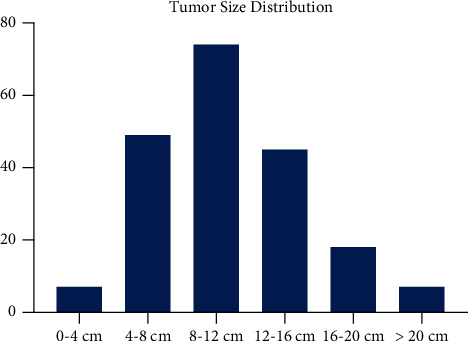
Tumor size histogram for osteosarcoma of the pelvis. The median tumor size was 11.0 cm and was the largest among surveyed anatomic sites.

Detailed measurements of tumor size were available for 200 cases. The median size of the tumors was 11.0 cm, with a range of 2.0 to 34.0 cm (Figure 2). There were 144 tumors greater than 8.0 cm (72.0%) and 56 tumors of 8.0 cm or smaller (28.0%).

Tumor extension was reported for 254 cases across all osteosarcoma subtypes, with 63 (24.8%) tumors contained to the originating bone versus 177 (69.7%) invasive or extracompartmental tumors, defined as expanding past the originating bone. Of the extracompartmental tumors, 112 were clearly defined in the database as extending through the periosteum, 55 invaded the adjacent bone or cartilage, and 10 demonstrated skip lesions. There was no significant difference between the invasiveness of conventional/osteoblastic osteosarcoma and chondroblastic osteosarcoma (*p*=0.308). However, the average tumor size of chondroblastic histology (125.7 mm) was significantly greater than of conventional histology (108.5 mm, *p*=0.038).

Data regarding the presence of metastasis at diagnosis were available for 275 pelvic osteosarcoma cases. Across all osteosarcoma subtypes, 92 (33.5%) cases presented with metastasis at diagnosis and 183 (66.5%) cases presented without evident metastasis. When the location of the metastasis was reported, the lung was the most common site, having involvement in at least 83.7% of the cases. Lymph node metastasis was noted in 9.4% of cases—usually associated with concurrent lung involvement. There was no statistical difference regarding presence of metastasis between osteosarcoma histologic subtypes and metastasis rate, nor was there a statistical difference between the presence of metastasis and the size of the tumor (*p* = 0.446 and *p* = 0.254, respectively).

Histologic grade was described for only 193 cases. The large majority of cases were described as high grade (94.3%), with only 5.7% of cases reported as low grade. The median age for low-grade cases was 44 years, compared to 48.5 years for high-grade cases, which was not significantly different. Of the 11 cases described as low grade, five had conventional/osteoblastic histology, four had chondroblastic histology, and one each had fibroblastic and intraosseous well-differentiated histology. There was no significant difference in tumor size between low- and high-grade cases. All low-grade cases were nonmetastatic on presentation, and 73% underwent surgical resection.

Information regarding surgical intervention of the primary tumor site was available for 289 cases of pelvic osteosarcoma, with 137 (47.4%) patients undergoing surgery and 152 (52.6%) patients reportedly not receiving any surgical intervention. Of the 127 surgical cases, 94 were treated with internal hemipelvectomy and limb salvage, while 7 were clearly treated with external hemipelvectomy/hindquarter amputation. Thirty-six patients had surgery, but the intervention could not be deduced from the codes, as they were coded as hemipelvectomy or surgery, not otherwise specified, leading to confusion as to whether these were internal or external hemipelvectomies. We therefore did not perform statistical assessment between types of surgical interventions, beyond assessing surgery on a “yes/no” categorical basis. Within these limitations, we could assess the association between surgery and several variables, including age, histologic subtype, tumor size, invasiveness, and presentation with metastatic disease. There was no association between the likelihood of having surgical intervention and histologic subtype, tumor size, or tumor invasiveness. There were significant differences, however, in the likelihood of surgical intervention based on age as well as presentation with metastatic disease. With regards to age, surgery was performed in 60.6% of patients <25 years of age and in 52.4% of patients who were 25–60 years of age, but only 27.1% of patients >60 years of age (*p* < 0.001 between age >65 and the age <25 and 25–60 groups). In cases where metastatic disease was present at the time of diagnosis, only 20.7% underwent surgical resection for local control, compared to 62.6% of patients having surgery when presenting without evident metastatic disease (*p*=0.010). When combining the variables of age and metastatic presentation, metastasis at diagnosis was associated with low percentages of surgical intervention, regardless of the age group. For those patients with metastasis at diagnosis, the percentage having surgery was 25.9% for <25 years, 21.9% for the 25–60 group, and 16.1% for >60 age group (*p*=0.578).

Information regarding the use of chemotherapy was available for 292 patients, but the data were not patent when assessed closely. The database identified 207 (70.9%) patients who received chemotherapy, while 85 (29.1%) had “unknown” or no use of chemotherapy. Effectively dividing these 85 patients into a group that clearly had no chemotherapy and a group with an unknown status could not be done within the confines of the database. Despite this limitation, division of patients who clearly received chemotherapy could be assessed by tumor size, metastatic disease, and age. There was no significant association between reported use of chemotherapy with tumor size or metastatic disease. However, there was a significant relationship between chemotherapy use and patient age. In patients under 25 years of age, 94.3% received chemotherapy, while 77.3% of patients aged 25–60 and 38.2% of those >60 had clearly reported treatment with chemotherapy (*p* < 0.001).

In a similar fashion, data regarding radiation use were “reported” for 292 patients, with 63 (21.6%) receiving radiotherapy and the remaining 229 patients (78.4%) with no use of radiation or “unknown” radiation status. Dividing this group of 229 patients into an unknown group and a “no treatment” group could not be clearly done, so statistical assessment was not performed for this subset.

### 3.2. Survival Rates of Primary Osteosarcoma of the Pelvis

**Figure 3:**
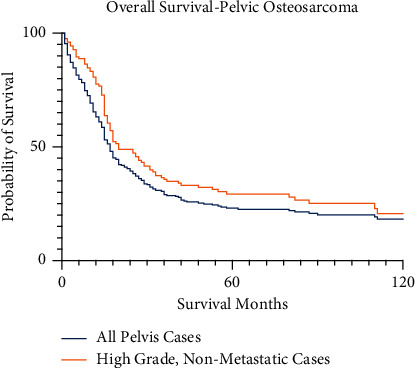
Kaplan–Meier survival curve for all pelvic osteosarcoma patients in this SEER cohort.

For the entire cohort of the SEER database from 2004 to 2015, the 2-, 5-, and 10-year survival rates for primary osteosarcoma of the pelvis are 45.6%, 26.5%, and 21.4%, respectively (Figure 3). In consideration of the confounding influence of metastasis and histologic grade, the 2-, 5-, and 10-year survival for high-grade pelvic osteosarcoma without metastasis at presentation was 55.7%, 35.5%, and 24.8%, respectively. The survival for the nonmetastatic, high-grade subgroup was not significantly different than the survival for the entire cohort (*p*=0.068). Survival outcomes for the entire pelvis cohort and subgroups are summarized in Table 1.

**Figure 4:**
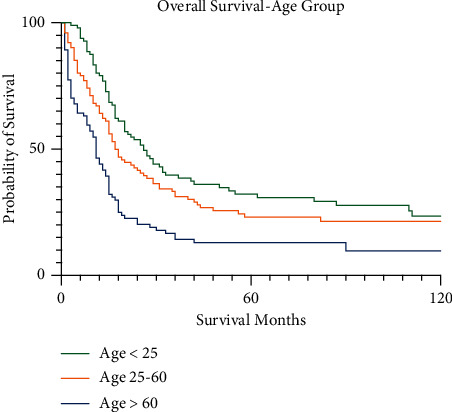
Kaplan–Meier survival curves for pelvic osteosarcoma patients by age group.

For pelvic osteosarcoma, the overall survival varied by age (Figure 4). Patients under the age of 25 had a 5-year survival of 34.3% (95% CI 24.3–44.5), compared to 25.0% (95% CI 15.9–35.1%) for patients aged 25–60 and 12.8% (95% CI 4.7–25.2%) for patients over the age of 60. These differences were not statistically different between under 25 and 25–60 age groups (*p* < 0.074), but survival for the over 60 age group was significantly worse than the other two age groups (*p* < 0.001).

The overall survival of chondroblastic osteosarcoma demonstrated statistically significant, better outcomes than conventional/osteoblastic osteosarcoma across all timepoints (*p*=0.019). When assessed by subtype, the 5-year survival for conventional osteosarcoma was 23.5% (95% CI 16.6–31.1%) versus 29.3% (95% CI 17.5–42.1%) for the chondroblastic osteosarcoma subtype. Data on the uncommon osteosarcoma subtypes, including fibroblastic, telangiectatic, and Paget-associated osteosarcoma, are scarce, and comparative survival statistical assessment could not be completed in this series due to the limited number of cases.

The overall survival of patients with tumors ≤8 cm was significantly greater than cases with tumors >8 cm (*p*=0.033). The 5-year survival rate for tumors ≤8 cm was 33.7% (95% CI 18.5–49.6%) compared to 27.0% (95% CI 19.0–35.7%) for tumors of size >8 cm. Local invasiveness of the tumor did not influence the overall survival in this cohort of patients.

**Figure 5:**
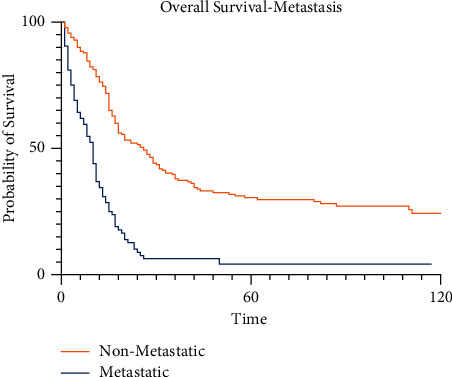
Kaplan–Meier survival curves for pelvic osteosarcoma patients by presence of metastatic disease.

The presence of metastatic disease at diagnosis had a profound, statistically significant impact on survival at all time points (*p* < 0.001). Cases of pelvic osteosarcoma diagnosed with evident metastatic disease at presentation had a 5-year survival of 5.3% (95% CI 1.3–13.7%) compared to the 5-year survival of 36.5% (95% CI 28.2–44.9%) for cases without diagnosed metastatic disease on presentation (Figure 5). No patients in the metastatic group survived to 10 years, whereas 28.6% of the group without metastatic disease at presentation survived to 10 years.

**Figure 6:**
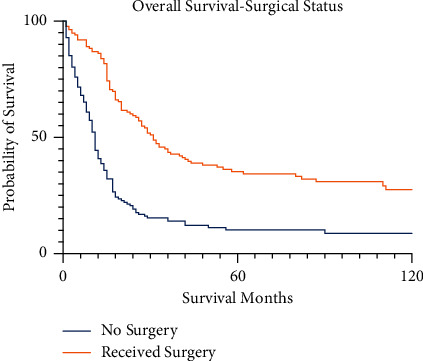
Kaplan–Meier survival curves for pelvic osteosarcoma patients by status of surgical intervention.

Patients treated with surgery fared better than those who did not receive surgery. The 5-year survival of the surgical group for pelvic osteosarcoma was 39.6% (95% CI 30.2–48.9%) versus 11.0% (95% CI 5.7–18.5%) for those who did not receive surgery (*p* < 0.001, Figure 6). Among patients with non-metastatic, high-grade tumors clearly defined as having received surgical resection and chemotherapy, the 5-year survival was 37.0% (95% CI 24.4–49.6%). Details regarding the survival of specific types of surgery are listed in Table 1, but there are significant constraints on interpreting statistical difference between surgical interventions due to coding ambiguity and the high proportion of cases listed as “hemipelvectomy, not otherwise specified.” The 5-year survival for patients definitively described as having received internal hemipelvectomy or a similar excisional procedure was 48.4% (95% CI 35.9–59.9%). The 5-year survival for patients receiving internal hemipelvectomy with high-grade tumors and no metastatic disease on presentation was 46.1% (95% CI 30.2–60.5%). Only 7 patients were explicitly described as having received external hemipelvectomy or hindquarter amputation, with a 5-year survival of 14.3% (95% CI 0.7–46.5%). Patients who underwent radiation in conjunction with surgery had a 5-year survival of 37.5% (95% CI 7.3–44.9%), while none of the patients who had radiation without surgery were alive at 5 years. These rates were not compared for statistical significance given the extent of absent data within the radiation reporting category.

A Cox proportional regression analysis was performed, which demonstrated older age, presence of metastatic disease at diagnosis, larger tumor size, and non-surgical status to be the most significant variables predicting worse overall survival. Sex, histologic subtype, and tumor invasiveness were not significant predictors of survival in this hazards model. Use of chemotherapy or radiation was not incorporated into the hazards model due to the unreliability of the data. These findings are summarized in Table 2.

### 3.3. Comparison of Pelvic Osteosarcoma to Osteosarcoma from Other Anatomic Sites

Between 2004 and 2015, the SEER database has data for a total of 292 cases of primary osteosarcoma of the pelvis, representing 9.8% of patients among 366 cases of primary upper extremity osteosarcoma, 1852 cases of the lower extremity, 79 cases of the vertebral column, 79 cases of the thorax, and 324 cases at the face/skull. The database yielded statistically significant higher rates of metastatic disease on presentation and worse survival for patients with primary osteosarcoma of the pelvis when compared to all other anatomic locations, except the spine (Table 3). The rate of metastatic disease on presentation varied among anatomic locations, such that 21.9% of upper extremity osteosarcoma was metastatic at diagnosis, compared to 18.7% for the lower extremity, 24.7% at the vertebral column, 19.7% at the thorax, and 4.2% at the face/skull bones, and the rate of metastasis at diagnosis was highest for pelvic tumors at 33.5%, which was significantly greater than the rate of metastasis at the upper/lower limbs and facial bones (*p* < 0.05). These data are summarized in Table 3.

**Figure 7:**
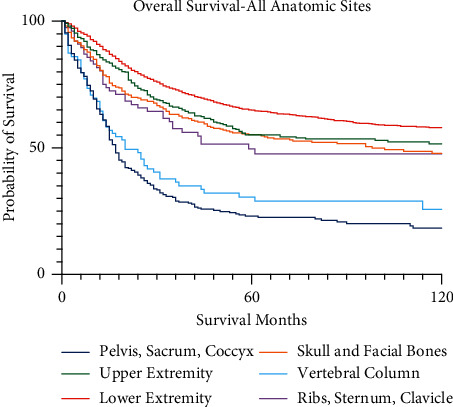
Kaplan–Meier survival curves for all osteosarcoma patients by primary anatomic site.

The 5-year survival for patients with upper extremity osteosarcoma was 57.7% (95% CI 52.0–63.0%), the lower extremity was 66.1% (95% CI 63.8–68.3%), the vertebral column was 33.0% (95% 21.5–44.8%), the thorax was 60.0% (95% CI 44.8–72.3%), and the face/skull was 62.5% (95% CI 56.1–68.6%; Figure 7). This compares to 26.5% 5-year survival of primary osteosarcoma of the pelvis (95% CI 20.5–32.8%). Each anatomic site was significantly different in survival when compared to the pelvis (*p* < 0.001), with the exception of the spine cohort.

## 4. Discussion

The primary purpose of this study was to investigate the presentation and outcomes of osteosarcoma of the pelvis as a distinct clinical entity. The secondary purpose of this study was to compare these outcomes to osteosarcomas from other anatomic sites. The data collected shed light on the nature of primary osteosarcoma of the pelvis and demonstrate meaningful, statistically significant differences in the prognosis of pelvic osteosarcoma when compared to other anatomic sites.

This study presents 292 pelvis osteosarcoma patients within the SEER database from 2004 to 2015. The timeframe was selected so that the Collaborative Staging variables (tumor size, metastasis, invasiveness) could be assessed, both in terms of their descriptive presenting characteristics and their influence on overall survival. Pelvic osteosarcoma, as demonstrated by the current assessment, tends to present as large, high-grade, invasive tumors with a high rate of metastatic disease on presentation. The factor that had the most profound influence on survival in this cohort of patients was the presence of metastasis at time of presentation. The 5-year survival for this subset of patients was 5.3%, and none of these patients survived to 10 years. Other factors that affected survival were age at diagnosis and the “eligibility” to be a surgical candidate.

Surgical intervention, when compared to no surgical intervention, is associated with improved survival in this study. While this result is unsurprising, given the totality of the medical literature dedicated to the treatment of osteosarcoma and this literature's support of wide surgical resection for osteosarcoma, interpretation of the surgical intervention in any cohort of pelvis osteosarcoma patients must be closely considered. Certainly, surgical resection is part of the gold standard treatment of osteosarcoma, and any regimen that does not routinely include wide surgical resection of osteosarcoma fails to meet this standard [6]. Without surgery, a resultant decline in survival is to be expected. Yet, in the current study, 52.6% of patients did not receive surgical resection of their tumor. Rather than representing a profound deviation from the gold standard of the participating institutions of the SEER registry, we infer and recognize that this large proportion of the cohort may have presented with tumors that were either deemed “unresectable” or associated with metastatic disease before the time of expected surgical intervention. Indeed, 51.8% of patients that did not have surgical intervention had metastatic disease on presentation, higher than the 33.5% metastatic rate noted for the entire cohort. However, this means that nearly half of the pelvic osteosarcoma patients who did not get surgery had no metastatic disease on presentation. Some other measure or clinical feature(s) were factored into the patients' and medical teams' decision to deem these tumors “unresectable.” Age was one of those factors in this series but does not fully explain the division of the cohort into surgical and non-surgical candidates. In fact, this data set did not demonstrate tumor size or local invasiveness to be different between the surgical and non-surgical groups.

The dataset does have significant limitations that limit a full exploration of the factors that went into the decision to have surgery or not. For example, some investigators in the literature list sacral extension of an osteosarcoma as a contraindication to surgery. The SEER database does not list this specific circumstance as a variable. While it may be that the shortcomings of the data set are responsible for not demonstrating significant differences between these two surgical groups in regard to the potentially relevant tumor variables, we suspect that there may be an element of a “gestalt” at play. That is to say, the resectability of a specific pelvis osteosarcoma is assessed by the totality of factors involved in the presentation, and, of the cases of pelvis osteosarcomas noted in the SEER database between 2004 and 2015, half presented with features that were deemed surgically unresectable—even when a dismal, subsequent prognosis was likely known to the patient and sarcoma medical team. In this sense, resectability of the tumor is, in itself, an important prognostic factor, even if the concept is difficult to precisely define within the limits of the database variables. Likewise, caution should be used to refrain from simply inferring that survival in pelvic osteosarcoma would necessarily be improved if only surgical intervention was done for lesions that would otherwise be deemed “unresectable.” Whenever possible, surgical resection should be advocated, and referral to a sarcoma center that specializes in such care is a must, but data demonstrating limited long-term survival also suggest that surgical procedures with undue morbidity may not be in the patient's best interest if the totality of the presentation would suggest a limited survival prognosis. Despite the limitations of the SEER data and potential coding inaccuracies and taking into account the influence of metastasis and patient age on the decision to undergo pelvic tumor resections, these data introduce the possibility that many patients in this review were surgically undertreated.

In reviewing the literature of studies dedicated to pelvic osteosarcoma, the 5-year survival rates reported in this study are similar to prior manuscripts [8, 15, 17–26]. Previously published, dedicated pelvis series have had between 19 and 121 patients, with overall 5-year survival rates of 13–34%. Based on the data from the current study, this range is likely due to the varying presenting features of the osteosarcoma and their influence on inclusion criteria for each manuscript. Isakoff et al. [18], for example, only included surgical candidates in their study and reported a 5-year survival rate of 38%, while Donati et al. [15] reported on all patients presenting with osteosarcoma, and they noted a 5-year survival of 15%. The 5-year survival rates for these cohorts of pelvic osteosarcoma are available in Table 4.

Comparison of the survival rates of osteoblastic and chondroblastic histology in the present study demonstrates significant and superior overall survival on univariate assessment for cases of pelvic osteosarcoma with chondroblastic histology—a result that runs in contradiction to previously published findings. Tsagozis et al. describe poor survival of chondroblastic osteosarcoma relative to osteoblastic osteosarcoma and suggest this is likely a result of poor response to chemotherapy and higher rate of metastasis with chondroblastic histology [26]. In fact, they describe the percentage of tumor necrosis to decrease in inverse proportion to the percentage of chondroblastic elements. Notably, the median age for chondroblastic histology in this cohort was 22 years, compared to 53.5 years for osteoblastic histology, and when survival outcomes were stratified by age, there was no significant difference in survival between histologic subtypes. Overall, this suggests patient age is a confounding variable for the unexpected survival rates observed between these two subtypes; therefore, this reported survival should be interpreted with caution. There would be benefit in exploring age differences relative to osteosarcoma histology in future large-scale studies.

In regard to surgical resection, the literature demonstrates the difficulties in obtaining negative (R0) margins in the resection of pelvis osteosarcoma. For example, Fahey et al. deemed that only 18 of their 25 pelvis osteosarcoma patients were surgical candidates and of the 18 surgical patients they treated, only 4 (22.2%) had wide, negative margins [24]. Similarly, Parry et al. attempted treatment as cure in 79 of their 121 patients, but only deemed 53 of these tumors resectable; they were able to achieve a wide resection in 18 of these 53 patients (34.0%) [22]. For Fuchs et al., an “adequate” margin was achieved in 30 of their 43 patients (69.8%) [22]. Likewise, Donati et al.reviewed 60 pelvic osteosarcoma cases; they deemed 30 cases resectable and, of the 30 patients who underwent surgery, obtained a wide, negative margin in 18 (60%) [21]. These reports illustrate the difficulties of the pelvis as a site of osteosarcoma: even when selecting patients for resectability, achieving a negative, wide surgical margin is difficult and often unpredictable.

In the second portion of our assessment of the SEER database, the overall survival was worse for osteosarcomas of the pelvis than other anatomic sites, with the exception of the spine. The reason for this worse outcome is likely multifactorial, but a profound influence on the poor prognosis of pelvic osteosarcoma is likely due to the increased rate of metastasis of pelvic osteosarcoma versus other sites and the number of pelvic tumors deemed unresectable by the treatment team. When comparing pelvic osteosarcoma to other anatomic locations of osteosarcoma, the pelvis tumors presented with a statistically significant higher rate of metastatic disease, with the exception of the spine. That being said, even in cases when metastasis was not present at time of diagnosis, the survival rates of pelvic osteosarcoma remained significantly worse than those rates reported in other non-spine anatomic sites. In this series, the 5-year survival of pelvis osteosarcoma without metastasis at diagnosis was 36.5%, which is worse than other anatomic sites, even when the data for those other sites include patients presenting with metastatic disease [4, 8–10]. This suggests that there are features inherent to pelvis osteosarcoma, beyond the higher rates of metastasis on presentation versus other anatomic sites, which make this location worse than other sites in the body. The data of this study and prior literature suggest these features may include differences in (1) age, (2) the rate of resectability, and (3) the rate of obtainable, negative surgical margins between pelvis osteosarcoma and other anatomic locations.

The limitations of this study are those inherent to any database study. The quality of data is dependent upon the input. Even if small, variations in treatments, follow-up, and reporting likely exist between the institutions participating in the SEER database. The influence of these variations must be acknowledged. In order to minimize the influence of changing treatment protocols and the quality of staging studies over time, we restricted our search to the years 2004–2015. In addition to the fact that treatment protocols during this timeframe have not substantially changed from current practice, this allowed us to query the Collaborative Stage variables for the tumor. Despite this limited and recent collection period, the data remained with gaps in reporting, which could affect the quality of the data. For example, 92 out of 292 pelvis osteosarcoma patients had no information on tumor size. Whether these missing data would alter the tumor size data reported is not known, as a nonreport could simply be random, or it could be associated with an unknown factor that is not found in the data. In addition, the codes in the SEER database can be left to interpretation, influencing the meaning of the data. Perhaps, the most striking example of this was found in the surgical intervention data. Surgical code 53 is listed as “Hemipelvectomy, NOS.” This could either be an internal or external hemipelvectomy. Thirty cases were coded with the 53 code, and other cases were similarly coded with unclear procedures, limiting the ability to interpret the nature of surgical interventions and their influence on outcomes. However, given the rare nature of osteosarcoma, and pelvis osteosarcoma in particular, this database study does provide an opportunity to assess a patient series that is larger than any single institution would be able to present in a reasonably narrow span of time.

## 5. Conclusion

The SEER database indicates pelvic osteosarcomas typically present as high-grade tumors, with a size greater than 8 cm, extension beyond the originating bone, high likelihood of metastatic disease at diagnosis, and a potential limited ability of disease to be addressed surgically. The survival rates of primary osteosarcoma of the pelvis are poor and demonstrate lower survival than primary osteosarcomas from other anatomic locations, in large part due to higher rates of metastatic disease and older age at presentation, larger tumor size, and lower rates of surgical resection compared to other anatomic sites. These findings clarify conditions affecting disease prognosis and highlight the importance of understanding appropriate criteria for oncologic resection of pelvic sarcomas.

## Figures and Tables

**Table 1:** Demographics and tumor characteristics for patients with primary osteosarcoma of the pelvis within the SEER database, 2004–2015, with 2-, 5-, and 10-year survival.

Variable		*n*	%	Survival			*p* ^*∗*^
				2-year	5-year	10-year	
All patients		292	100	45.6	26.5	21.4	
Sex	Male	149	51.0	43.3	26.8	21.8	0.481
	Female	143	49.0	48.0	26.1	20.9	
Age group	Age <25	96	32.9	54.0	34.3	25.0	<0.001
	Age 25–60	104	35.6	44.9	25.0	23.1	
	Age >60	92	31.5	28.2	12.8	12.8	
Histology	Osteoblastic	204	69.9	41.6	23.5	20.0	0.074
	Chondroblastic	65	22.3	55.5	29.3	26.0	
	Paget-associated	6	2.1	25.0	0	0	
	Other	17	5.8	53.8	46.2	28.8	
Tumor size	Tumor ≤8 cm	56	28.0	58.3	33.7	30.3	0.033
	Tumor >8 cm	144	72.0	42.1	27.0	18.1	
Tumor extension	Contained to the originating bone	63	24.8	52.3	31.7	28.5	0.550
	Extension beyond the periosteum	177	69.7	42.9	25.2	18.6	
	Infiltrating the adjacent bone or cartilage	55	21.7	39.5	23.7	14.2	
	Skip metastasis	10	3.4	50.0	50.0	50.0	
Metastasis	None at presentation	183	66.5	59.7	36.5	28.6	<0.001
	Metastasis at any location	92	33.5	12.7	5.3	0	
	Metastasis to lung only	41	14.9	20.5	5.1	0	
	Metastasis to lung and other nonlymph node site	36	13.1	7.4	7.4	0	
Surgery	None	152	52.6	23.4	11.0	11.0	<0.001
	Any surgery	137	47.4	65.1	39.6	30.4	
	Internal hemipelvectomy/excision	94	32.5	68.7	48.4	37.5	
	External hemipelvectomy	7	2.4	57.1	14.3	0	
	Unspecified surgical procedure	36	12.5	59.4	28.1	20.1	
Radiation	Received radiation and surgery	19	30.2	60.0	37.5	37.5	
	Received radiation without surgery	44	69.8	16.7	0.0	0.0	

^*∗*^ *p* values are for Kaplan–Meier log-rank tests for pooled variables in each subcategory.

**Table 2:** Cox proportional hazards model.

Variable	Hazard ratio	95% confidence interval	*p*
Age <25 (reference)			
Age 25–60	1.641	1.079–2.495	0.020
Age >60	2.358	1.440–3.859	<0.001
Male (reference)			
Female	1.034	0.732–1.461	0.850
Osteoblastic histology (reference)			
Chondroblastic histology	0.705	0.461–1.078	0.107
Intracompartmental (reference)			
Extracompartmental	1.062	0.655–1.724	0.806
Nonmetastatic (reference)			
Metastatic at diagnosis	2.839	1.845–4.367	<0.001
Tumor ≤8 cm (reference)			
Tumor >8 cm	1.541	1.017–2.336	0.042
No surgery (reference)			
Received surgery	0.551	0.365–0.831	0.004

**Table 3:** Comparison of pelvis osteosarcoma to other anatomic locations and overall survival at 2, 5, and 10 years.

Anatomic site	*n*	%	Median age at diagnosis (years)	Median tumor size (cm)	Metastasis at diagnosis (%)	Received surgery (%)	Survival		
							2-year	5-year	10-year
Pelvis	292	9.8	47.5	11.0	33.5	47.4	45.6	26.5	21.4
Upper limb	366	12.2	19.0^*∗*^	9.5^*∗*^	21.9^*∗*^	84.9^*∗*^	76.4^*∗*^	57.7^*∗*^	54.3^*∗*^
Lower limb	1852	61.9	17.0^*∗*^	9.2^*∗*^	18.7^*∗*^	88.4^*∗*^	81.0^*∗*^	66.1^*∗*^	59.3^*∗*^
Face and skull	324	10.8	43.5	4.5^*∗*^	4.2^*∗*^	89.2^*∗*^	75.1^*∗*^	62.5^*∗*^	54.5^*∗*^
Vertebral column	79	2.6	53	5.5^*∗*^	24.7	70.9^*∗*^	53.1	33.0	30.9
Thorax	79	2.6	46	6.8^*∗*^	19.7	81.0^*∗*^	73.6^*∗*^	60.0^*∗*^	57.0^*∗*^

Asterisk (^*∗*^) denotes difference from the pelvis at significance level <0.05.

**Table 4:** Previously published manuscripts describing 5-year overall survival of osteosarcoma of the pelvis.

Study	Years reviewed	*n*	5-year overall survival	5-year overall survival—surgical candidates
Current study	2004–2015	292	26.5%	39.6%
Parry et al.	1983–2014	121	27.2%	—
Donati et al.	1978–1998	60	15%	30%
Fahey et al.	1967–1990	25	20%	—
Fuchs et al.	1983–2003	43	—	38%
Grimer et al.	1971–1996	36	18%	41%
Ham et al.^*∗*^	1978–1995	40	26%	35%
Isakoff et al.^*∗*^	1993–2005	26	—	38%
Kawai et al.	1977–1994	40	34%	41%
Ozaki et al.	1979–1998	67	27%	41%
Saab et al.	1970–2004	19	26%	—
Song et al.	1990–2006	41	29%	56%

^*∗*^Survival for stage IIB only—stage III was excluded in survival analysis.

## Data Availability

The data used in this study are found in the publically available Surveillance, Epidemiology, and End Results database and are available for independent review.
